# An ovine tracheal explant culture model for allergic airway inflammation

**DOI:** 10.1186/1476-9255-7-46

**Published:** 2010-08-30

**Authors:** Latasha Abeynaike, Els NT Meeusen, Robert J Bischof

**Affiliations:** 1 Biotechnology Research Laboratories, Department of Physiology, School of Biomedical Sciences, Monash University, Clayton VIC 3800, Australia

## Abstract

**Background:**

The airway epithelium is thought to play an important role in the pathogenesis of asthmatic disease. However, much of our understanding of airway epithelial cell function in asthma has been derived from *in vitro *studies that may not accurately reflect the interactive cellular and molecular pathways active between different tissue constituents *in vivo*.

**Methods:**

Using a sheep model of allergic asthma, tracheal explants from normal sheep and allergic sheep exposed to house dust mite (HDM) allergen were established to investigate airway mucosal responses *ex vivo*. Explants were cultured for up to 48 h and tissues were stained to identify apoptotic cells, goblet cells, mast cells and eosinophils. The release of cytokines (IL-1α, IL-6 and TNF-α) by cultured tracheal explants, was assessed by ELISA.

**Results:**

The general morphology and epithelial structure of the tracheal explants was well maintained in culture although evidence of advanced apoptosis within the mucosal layer was noted after culture for 48 h. The number of alcian blue/PAS positive mucus-secreting cells within the epithelial layer was reduced in all cultured explants compared with pre-cultured (0 h) explants, but the loss of staining was most evident in allergic tissues. Mast cell and eosinophil numbers were elevated in the allergic tracheal tissues compared to naïve controls, and in the allergic tissues there was a significant decline in mast cells after 24 h culture in the presence or absence of HDM allergen. IL-6 was released by allergic tracheal explants in culture but was undetected in cultured control explants.

**Conclusions:**

Sheep tracheal explants maintain characteristics of the airway mucosa that may not be replicated when studying isolated cell populations *in vitro*. There were key differences identified in explants from allergic compared to control airways and in their responses in culture for 24 h. Importantly, this study establishes the potential for the application of tracheal explant cultures in relevant *ex vivo *investigations on the therapeutic and mechanistic modalities of asthmatic disease.

## Background

Asthma is a complex chronic inflammatory disease, the hallmarks of which include damage to the airway epithelium and underlying parenchyma, recruitment and activation of inflammatory cells, and airflow obstruction associated with remodeling of the airways. The mucosal epithelial layer of the airways plays a pivotal role in the non-specific host defense of the respiratory tract and in shaping both innate and adaptive immune responses of the respiratory system [1,2]. In asthma, the hypertrophy of submucosal glands and hyperplasia of goblet cells in the airways [3] form the basis of excessive mucus production, leading to sometimes fatal bronchial plugging. Furthermore, many of the pathological features of asthmatic tissues such as tissue injury and inflammation are triggered in part by mediators derived from the airway epithelium [1,4].

*In vitro *cell-based studies have allowed detailed investigations of the molecular mechanisms underlying the pathology of asthmatic disease. This has included studies in humans and appropriate animal models using primary airway epithelial, fibroblast and smooth muscle cells cultured from biopsy samples collected by fibreoptic bronchoscopy or post-mortem tissues [5,6]. However, an inherent limitation of these *in vitro *investigations is the inherent loss of tissue-specific cell differentiation and tissue architecture that is associated with studying isolated cells, and particularly cells of epithelial origin [6]. The development of more appropriate cell/tissue culture systems for airway epithelial investigation, that uses an air-liquid interface, has demonstrated differentiation into mucus-secreting and ciliated cells that display key functional differences compared to cells grown in submerged cultures [7,8]. In addition, the growth of explant cultures established from tracheal tissues adds a further dimension in providing the opportunity to examine *ex vivo *the interactions between epithelium and underlying structural cells of the airway mucosa. Tracheal explants established in sheep display many key features of the *in vivo *airways such as mucus coverage, mucociliary clearance and cell structure [9]. While such studies have been used to examine approaches for gene delivery [9-11] and mechanisms of epithelial cell mucus secretion [12], none to date have used tracheal explants to investigate the allergic basis of bronchial asthma. In this study we use a validated model of human allergic asthma [13-15] to investigate airway mucosal responses *ex vivo *in tracheal explants derived from normal sheep and allergic sheep exposed to house dust mite (HDM) allergen.

## Methods

### Experimental sheep, allergen sensitization and airway challenges

Female Merino-cross ewe lambs (4-5 months of age) free of significant pulmonary disease [14] were used for these studies. All experimental animal procedures and the collection of tissues and cells were approved by the Animal Ethics Committee of Monash University, following guidelines set by the National Health and Medical Research Council (NH&MRC) of Australia.

Sheep were sensitized by immunization with solubilized HDM whole extract (*Dermatophagoides pteronyssinus*), and immunized sheep were classed as allergic when they showed increased HDM-specific serum IgE levels as assessed by ELISA [14]. Naïve, control animals were not immunized with HDM. All allergic sheep were subsequently given 3 airway allergen challenges at weekly intervals to prime the respiratory tract to HDM allergen [16,17] and BAL samples were collected using a fibre-optic bronchoscope to assess airway inflammation [14]. The allergen challenges were followed by a rest period of 2 weeks before all animals were euthanased (barbituate overdose) for the collection of tracheal tissues.

### Preparation of t**racheal explant cultures**

Following resection, the trachea was cut longitudinal along the midline through the trachealis, and pinned open onto a dissecting board. The mucosa was dissected gently away from cartilage and small 5 mm discs of tracheal tissue were cut away using a biopsy punch (Kai Medical, Germany). Tracheal explants were established in culture as outlined previously [9], with some modifications. Briefly, sterile strips of filter paper (Whatman^® ^Grade 1; Sigma, Australia) pre-soaked in medium (DMEM10; DMEM with 10% fetal bovine serum + penicillin/streptomycin; Invitrogen, Life Technologies, Australia) were placed across a 35 mm diameter culture dish sitting within a larger 60 mm dish, to which 3 ml DMEM10 was added. For each treatment, triplicate tracheal biopsies were placed upon the strip of filter paper set up within each plate.

To investigate possible changes in epithelial morphology and tissue architecture, tracheal biopsies were collected from control (naïve) sheep and explants prepared in triplicate as detailed above. Following the application of 5 μl of DMEM containing HDM allergen (10 μg/ml) or 5 μl of control DMEM to the epithelial surface, explants were cultured for a period of 5 h, 24 h or 48 h at 37°C in 5%CO_2_/air. In a separate experiment, tracheal tissues were resected from allergic and control (naïve) sheep and tracheal explants established in triplicate cultures for a 24 h period.

### Tissue processing and histology

After culture, tracheal explants were transferred into a small tube containing 100 μl DMEM10, briefly agitated and (medium washout) samples stored at -20°C for later cytokine analyses. Tissues were then fixed in 4% paraformaldehyde (PFA) and embedded in paraffin for histology. Paraffin-embedded tracheal tissue explants were sectioned (7 μm) and stained with hematoxylin and eosin (H&E), alcian blue/periodic acid-schiff (AB/PAS) or immunostained to identify caspase-3 positive apoptotic cells, mucosal mast cells and eosinophils.

### Immunohistochemistry for caspase-3, mast cells and eosinophils

Immunostaining for caspase-3 was used to identify apoptotic cells [18] in tracheal tissues before and after 5 h, 24 h and 48 h in culture. Briefly, paraffin-embedded PFA-fixed tissue sections were dewaxed, then rehydrated and immersed in 0.1 M PBS containing 0.3% triton X100 (PBS-TX) followed by 0.1 M citric acid buffer and microwave treatment for antigen retrieval. Sections were washed in PBS-TX, endogenous peroxidase activity blocked upon incubation with 0.3% H_2_O_2_, again washed and incubated with 5% normal goat serum (NGS)/2% BSA in PBS-TX. Sections were then incubated with rabbit polyclonal anti-human/mouse activated caspase-3 antibody (Ab) (R&D Systems, USA), washed and incubated with biotinylated goat anti-rabbit IgG (Cayman Chemical, USA), and again washed prior to incubation with streptavidin-horseradish peroxidase (HRP) complex (Amersham Biosciences, UK). Following washing, sections were developed with 3,3'-diaminobenzidine tetrahydrochloride (DAB; Sigma) and finally dehydrated and cover-slipped with Depex™ tissue mounting fluid (Fluka Biochemika, Switzerland).

For the detection of mast cells and eosinophils, paraffin embedded tissue sections were blocked as above, then incubated with 10% normal sheep serum in PBS prior to application of primary antibodies. A polyclonal rat anti-ovine tryptase Ab reactive with all ovine respiratory mast cells [19] was kindly provided by Professor Hugh Miller, University of Edinburgh, UK. For eosinophil detection, the ovine eosinophil-specific mouse anti-galectin-14 mAb (clone EL1.2) was used [20]. Following incubations, sections were washed then incubated with HRP-conjugated rabbit anti-rat Ig (Dako, Denmark) or HRP anti-mouse (Dako) for the detection of mast cells and eosinophils, respectively. This was followed by further washes, then development in DAB as detailed above. Slides were air-dried and lightly counterstained with Wright's stain (Sigma), then cover-slipped in Depex™.

### Cell counts

In AB/PAS stained sections, intensely purple staining cells within the epithelium (goblet cells) were enumerated in triplicate samples at 200× magnification using a calibrated grid. All intact tissue was examined for each treatment (maximum 15 microscopic fields) and counts were expressed as cell number per mm length of epithelium.

Caspase-3 positive cells, within the epithelial layer only, were enumerated in triplicate samples at 200× magnification using a calibrated grid. All intact tissue was examined for each treatment (maximum 15 microscopic fields) and counts were expressed as cell number per mm length of epithelium.

For mast cell and eosinophil cell counts, immunoperoxidase positive cells in the lamina propria underlying the epithelium were enumerated in triplicate samples at 200× magnification using a calibrated grid. All intact tissue was examined for each treatment (maximum 15 microscopic fields) and counts were expressed as cell number per mm^2 ^area of lamina propria underlying the epithelium. Cell counts for both mast cells and eosinophils were performed on cells that were densely stained. Cells partially stained were classified as degranulated and were not counted.

### Detection of cytokines in tracheal washout samples

Tracheal explant washout samples were collected for determination of the ovine cytokines IL-1α, IL-6 and TNF-α by enzyme-linked immunosorbent assay (ELISA), as described previously [21]. The minimum detectable levels of IL-6 and TNF-α were 2.63 ng/ml and 1.98 ng/ml, respectively.

### Statistical analyses

Results are presented as means ± SEM. A Kruskal-Wallis non-parametric test was used to compare treatments, within experimental and control groups, and a Dunn's post-hoc test was used where significant. A Mann-Whitney non-parametric test was used for comparisons between experimental and control groups. For all statistical analyses, *p *< 0.05 was considered significant.

## Results

Priming of the airways with allergen induced a marked recruitment of eosinophils (15-32% of BAL leukocytes) into the BAL fluid when assessed at 48 h post-challenge, similar to that observed in our previous studies [14,16].

### Histological and functional changes in tracheal explant cultures

**Figure 1:**
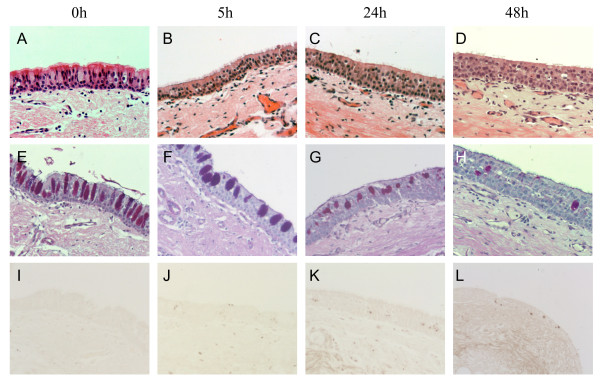
**Histology of tracheal tissues before and following culture *ex vivo***. **(A-D) **H&E stain demonstrating intact ciliated epithelium over a 48 h incubation period; little change in tissue integrity is seen at 48 h (D). **(E-H) **AB/PAS stain showing the presence of mucus-producing goblet cells; strong staining is evident in pre-cultured tissue (E), while a progressive decrease in staining is seen following 48 h of culture (H). **(I-L) **Caspase-3 immunostained sections demonstrating apoptosis; positive staining cells present in the epithelium and underlying tissue. Both cytoplasmic and nuclear staining is seen, with more intense nuclear staining evident at 48 h (L) (original magnification ×200).

Tracheal tissues were collected from control (naïve) animals to assess the effects of maintaining tracheal explants in culture for 0 h, 5 h, 24 h, and 48 h. Examination of the explants under a dissecting microscope throughout the culture period showed that the tracheal epithelium appeared intact, with evidence of actively beating cilia (not shown). This was supported in the histological examination of tracheal explant tissue sections; while there were several areas showing a less organised arrangement of cells and a reduced number of ciliated cells, there appeared to be little change in epithelial structure and general integrity of the cultured mucosal tissue compared to fresh pre-cultured (0 h) tissue (Figure 1A-D).

**Figure 2:**
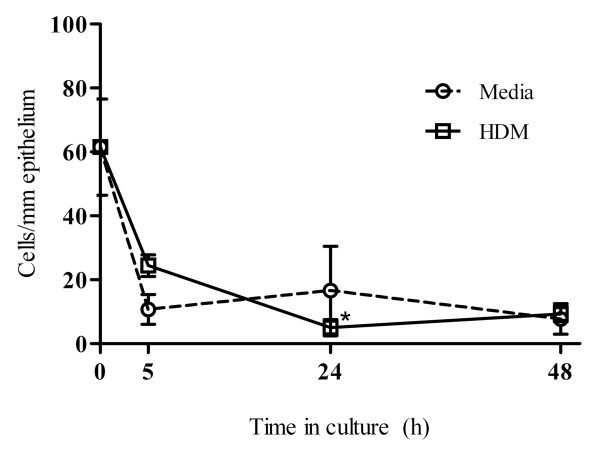
**Kinetics of goblet cells in the epithelium of control tracheal explants over a period of 48 h in culture**. Mean (± SEM) counts (triplicate cultures from n = 3 naïve sheep) of AB/PAS-stained goblet cells within the tracheal epithelium from cultures grown in media alone or in the presence of HDM allergen (* denotes statistical significance, p < 0.05; Kruskal-Wallis, comparison to 0 h pre-cultured tissue).

AB/PAS staining of sections revealed the presence of mucus-producing goblet cells within the tracheal explants. Positively stained goblet cells were seen in all sections, with staining particularly prominent in the tracheal epithelium prior to culture, followed by a dramatic reduction in the level of staining throughout the culture period (Figure 1E-H). Cell counts confirmed a rapid loss or decline in AB/PAS reactive goblet cells over time in both the media and HDM-treated tissue (Figure 2).

Immunostaining for caspase-3, a marker for cell apoptosis, was examined in paraffin sections of tracheal explants from control animals before and after culture. Caspase-3 staining of apoptotic bodies was seen in all sections of cultured tissue and staining was evident within the epithelium and underlying lamina propria (Figure 1I-L). No positive staining within the epithelium was seen in tissue at 0 h, while some staining was present in the underlying lamina propria. Staining of apoptotic cells was most prominent in tracheal explants cultured for 48 h when positive staining was seen in the nucleus as well as the cytoplasm, with intense nuclear staining representing more advanced apoptosis apparent in the epithelium compared to the earlier time-points (Figure 1I-L).

### Comparison of tracheal explants established from allergic and control tissues

Tracheal tissues were collected from allergic and control (naïve) sheep. The airways of allergic animals had been 'primed' with three aerosolised HDM challenges, resulting in the induction of airway eosinophilia and raised allergen-specific IgE levels in serum and bronchoalveolar lavage (BAL), as noted previously [14]. Two weeks after the last challenge, allergic and control animals were sacrificed for the collection of tissues, and tracheal explants were established in culture for a period of 24 h.

**Figure 3:**
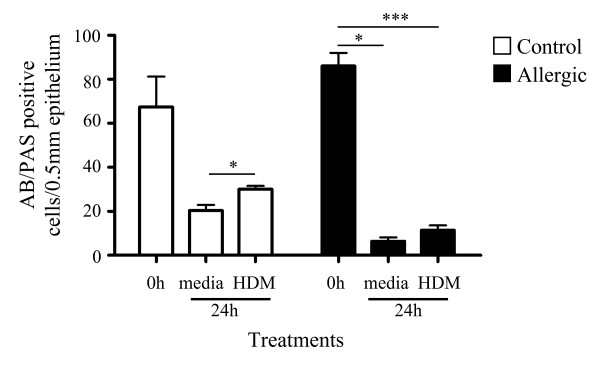
**Goblet cell numbers before and after culture in tracheal explants from control and allergic tissues**. AB/PAS positive cell counts (mean of triplicate cultures ± SEM) per 0.5 mm of epithelium in tracheal explants before (0 h) and following (24 h) culture in the presence of media alone or HDM. (* denotes statistical significance, *p *< 0.05, ***p < 0.001; Kruskal-Wallis).

At 0 h there were intense, AB/PAS-stained goblet cells in the epithelium as reported earlier (see Figure 1E-H), and the mean number of goblet cells was greater in allergic versus control epithelium (86.0 ± 6.1 vs 67.0 ± 13.9 cells/mm epithelium) although this difference was not statistically significant. A dramatic loss in goblet cell staining was observed after 24 h culture in medium alone or in the presence of HDM allergen (Figure 3), with a significantly greater percentage loss in allergic (86.9 ± 2.19) compared to control (60.61 ± 8.50) tissues (Mann Whitney; p = 0.036; percentage loss not shown in Figure).

**Figure 4:**
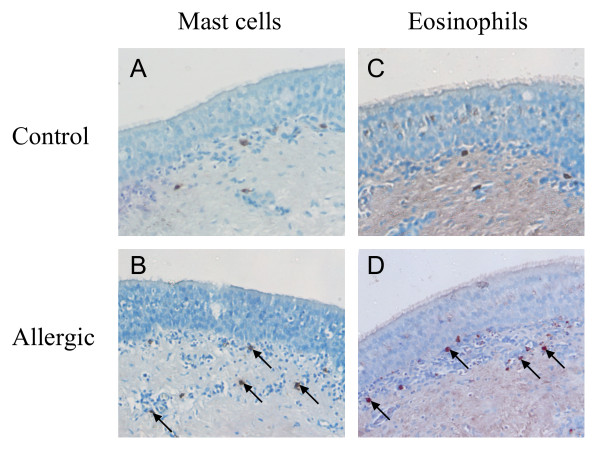
**Immunostaining for mast cells and eosinophils in tracheal explants**. Mast cell (tryptase^+^) staining in **(A) **control and **(B) **allergic tissues, and eosinophil (galectin-14^+^) staining in **(C) **control and **(D) **allergic tracheal explants. Note the increased number of immunopositive mast cells and eosinophils in the allergic (indicated by arrows in B and D) compared to the control tracheal explants (original magnification ×200).

**Figure 5:**
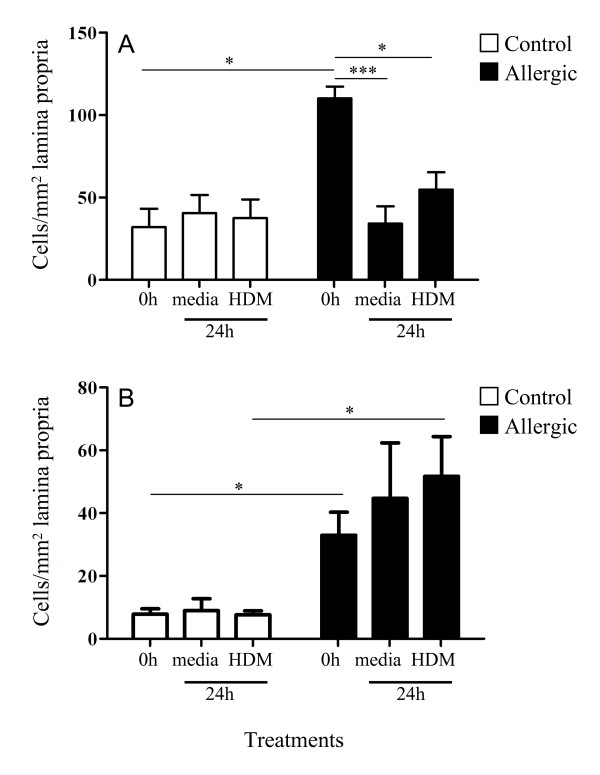
**Quantitation of mast cells and eosinophils in control and allergic tracheal explants**. Mean (± SEM) number of **(A) **mast cell and **(B) **eosinophil counts per mm^2 ^lamina propria in tracheal explants from control and allergic tissues prior to culture (0 h) or following 24 h culture in the presence of media alone or HDM. (* denotes statistical significance, *p *< 0.05, ***p < 0.001; Kruskal-Wallis for within-group comparisons; Mann-Whitney for allergic vs control).

Immunohistochemistry revealed that tracheal tryptase-positive mast cells were distributed throughout the lamina propria underlying the epithelium, but not localised within the epithelium (Figure 4A-B). Quantitation of tissue mast cells showed that prior to explant culture (0 h), mast cells were more numerous in allergic compared to control tissues (Figure 5A; p < 0.05). Following 24 h culture in medium with or without HDM, there was a significant loss of mast cell staining in the allergic tracheal explants to levels similar to that seen in the control tissues (Figure 5A). Large, diffusely-stained cells were typically seen in the allergic explant tissues, compared to smaller-stained mast cells in control tissues.

Galectin-14 positive eosinophils were localised mainly beneath the epithelial basement membrane, and were particularly prominent in allergic tissues (Figure 4C-D). Approximately five-fold greater eosinophil numbers were seen in pre-cultured (0 h), and HDM-treated tissue in allergic compared to control tissues (Figure 5B; p < 0.05). No change in eosinophil numbers was observed after 24 h culture in the absence or presence of HDM.

### Cytokine release by tracheal explants following HDM stimulation

ELISAs were performed on tissue washout samples for the soluble cytokines IL-1α, IL-6 and TNF-α. We were unable to detect TNF-α or IL-1α in any of the washout samples (data not shown), as they were below the detection limit for these assays.

IL-6 secretion was not detected in any of the control tracheal tissues (< 2.65 ng/ml), but was clearly evident in the washout samples from allergic tracheal explants cultured for 24 h in the presence of media alone (4.08 ± 0.67 ng/ml) or with HDM allergen (3.71 ± 1.20 ng/ml; not statistically significant).

## Discussion

The use of *in vitro *models, in particular primary cell cultures, has greatly contributed to our understanding of molecular pathways in allergic inflammation. However, asthma is a complex disease, and *in vitro *studies that focus on isolated cell populations may not accurately represent *in vivo *conditions where dynamic and complex interactions take place between different structural and inflammatory cells. Tissue explant cultures established *ex vivo *have been used for a variety of applications, and while their use may be restricted to short-term studies, they offer a distinct advantage in providing a more complete tissue microenvironment for experimentation that preserves many of the *in vivo *tissue characteristics. Previous studies in sheep have reported the use of tracheal explants for cystic fibrosis research [9,11]. The present study used a relevant large animal model of human asthma to establish for the first time an *ex vivo *tracheal explant model as an investigative tool for asthma research.

We demonstrate in the present study that, other than the dramatic loss of goblet cell numbers, which likely reflects a tissue response to the new (*ex vivo*) conditions rather than being solely indicative of tissue injury and cell death, the epithelial structure and general morphology of cultured tracheal explants was maintained for up to 48 h in culture. This is in agreement with an earlier study using this model system which reported that explants maintained over this period in culture clearly display cilia and microvilli (as assessed by scanning electron microscopy and light microscopy) as well as ciliary beating, retain normal barrier functions including mucociliary clearance and mucus production, and an intact cellular structure [9]. It should be noted that in the present study, however, apoptosis within the tracheal epithelium was already observed after 5 h in culture and, while there was no further increase in the degree of apoptosis with culture time, intense caspase-3 immunostaining of tracheal epithelial cells representing late stage apoptosis [18] was most evident after 48 h of culture.

One of the distinctive features of the asthmatic airways is mucus hypersecretion, associated with goblet cell hyperplasia and hypertrophy [3]. In this study, numbers of mucus-containing (AB/PAS positive) goblet cells in the tracheal epithelium of allergic sheep was similar compared to control sheep. Following 24 h in culture, lower numbers of goblet cells were observed in both allergic and control tissue, although this difference was significant only in the allergic tissues. This rapid decline is indicative of the release of mucus or a shutdown in mucus production and suggests that allergic tissue is particularly sensitive to the release of mucus. Alternatively, it is possible this may represent part of a stress response *in vivo *[22]. While there was a loss of goblet cell staining in culture, the effects of tissue processing for histological analyses most likely resulted in the loss of the mucus layer overlying the epithelium.

Mast cells are required for the development of allergic reactions, with cross-linking of the high affinity receptors for IgE resulting in degranulation and release of cytokines. This cross-linking is generally achieved by binding of allergen to allergen-specific IgE captured by the mast cells. Studies have shown increased mast cells within sheep alveolar septa and airway walls following chronic airway allergen (HDM) challenge [23]. In the present study mast cells were abundant in all tracheal tissues at 0 h, while there was a significant decline in mast cell numbers following culture in medium alone or in the presence of HDM in the allergic but not control tissues. Together with the more dramatic decline in goblet cells in the allergic tissue this may reflect the hypersensitive nature of the allergic explants. Further to this, mast cell staining in all tracheal explants from allergic tissue showed larger, diffusely-stained cells indicative of degranulating mast cells, compared to the smaller stained cells seen in control tissues. That HDM had no impact on mast cell numbers in either allergic or control explants is an intriguing observation but not unlike what we have previously observed in allergen-challenged dermal tissue [17]. It is possible that HDM allergens have an impact on other mast cell related activities, not examined in the present study, such as release of cytokines [24,25].

Airway eosinophilia is a central component of asthma pathogenesis. Upon recruitment to the airways, eosinophil activation results in the release of mediators and toxic granule proteins, the actions of which include epithelial damage and stimulation of mucus secretion [26,27]. In previous studies we reported a 4 to 6-fold increase in eosinophils in the lungs of HDM challenged sheep compared to control tissue [14]. In the present study, an approximate 5-fold increase in (galectin-14 positive) eosinophils was seen in allergic compared to control tissues. Increased galectin-14 has also been demonstrated by Western blot analyses in BAL fluid following airway allergen challenge in allergic sheep [20].

The secretion of the cytokines IL-6, TNF-α and IL-1α in the tracheal explant cultures was assessed by ELISA, and while TNF-α and IL-1α were undetected, detectable levels of IL-6 were released by allergic tissues but absent in control tissues. A previous study of IL-6 mRNA expression in human airway epithelial cells observed very low or no IL-6 levels in non-asthmatic tissue compared to asthmatic tissue [28]. IL-6 levels are significantly increased in asymptomatic asthmatic patients compared to control patients, as well as during naturally occurring asthma attacks [29], indicating involvement of IL-6 in asthma pathogenesis. Upregulation of IL-6 is also detected within airway epithelial cells in symptomatic asthma patients [28]. TNF-α and IL-1α on the other hand are predominantly produced in respiratory mucosa by tissue macrophages, perhaps explaining why they were undetected in the washout samples. Furthermore, cytokines released by the epithelium may be trapped in the mucus layer or may not be released apically.

Animal experimental models have proven to be imperative in understanding disease processes and recent research has focused in particular on the inflammatory mediators in the airways, in the hope of developing more specific and effective therapeutic avenues for asthma treatment. This study examined a tracheal tissue explant model as an investigative tool in asthma research. A distinct advantage of this explant model, compared to other *in vitro *models, is the preservation of the tissue architecture and microenvironment that may better reflect conditions and effects encountered *in vivo*. The present studies suggest this tracheal explant model may be most relevant for investigations over shorter culture periods (< 24 h), since tissue changes became more evident over longer periods in culture. As allergen stimulation causes an immediate hypersensitivity response in asthmatic subjects following degranulation of mast cells and release of inflammatory mediators, it would be interesting to examine in more detail the early time points after HDM challenge of tissue explants and measure the release of these mediators during this period in both allergic and non-allergic explants. Using a validated large animal model with similar airway structures to human, the tracheal explant model offers great potential for studying the complex mechanisms driving allergic disease and could be used as a platform for the screening of potential anti-asthma therapeutics.

## Competing interests

None of the authors has a financial relationship with a commercial entity that has an interest in the subject of this manuscript.

## Authors' contributions

LA carried out the majority of the experimental work and data analysis and drafted the manuscript. RJB developed the culture model and with ENTM conceived the study and participated in the experimental design, data analysis and manuscript preparation. All authors read and approved the final manuscript.
